# COVID-19 Vaccination Actual Uptake and Potential Inequalities Due to Socio-Demographic Characteristics: A Population-Based Study in the Umbria Region, Italy

**DOI:** 10.3390/vaccines11081351

**Published:** 2023-08-09

**Authors:** Chiara Primieri, Manuela Chiavarini, Irene Giacchetta, Chiara de Waure, Carla Bietta

**Affiliations:** 1 Local Health Unit 1 of Umbria, Prevention Department, Epidemiology Service, 06126 Perugia, Italy; chiara.primieri@uslumbria1.it (C.P.); carla.bietta@uslumbria1.it (C.B.); 2 Department of Biomedical Sciences and Public Health, Section of Hygiene, Preventive Medicine and Public Health, Polytechnic University of the Marche Region, 60121 Ancona, Italy; m.chiavarini@staff.univpm.it; 3 Department of Medicine and Surgery, University of Perugia, 06123 Perugia, Italy; irene.giacchetta@gmail.com

**Keywords:** COVID-19, inequalities, vaccination

## Abstract

Socio-demographic factors are responsible for health inequalities also in vaccination. The aim of this study was to evaluate their role at the population level through a population-based study performed on the whole population entitled to receive COVID-19 vaccines in the Umbria Region, Italy, and registered to the Regional Healthcare Service as of 28 February 2021. Socio-demographic characteristics and vaccination status in terms of uptake of at least one dose of any available vaccine, completion of the primary vaccination cycle and uptake of the booster doses as of 28 February 2022 were collected from the Umbria regional database. The percentage of eligible population who did not initiate the COVID-19 vaccination, complete the full vaccination cycle and get the booster dose was 11.8%, 1.2% and 21.5%, respectively. A younger age, being a non-Italian citizen, and not holding an exemption for chronic disease/disability and a GP/FP were associated with all the endpoints. Females, as compared to males, were more likely to not initiate the vaccination but less likely to not receive the booster dose. On the contrary, the findings did not show a significant association between the deprivation index and the vaccine uptake. The findings, beyond confirming current knowledge at the population level, provide new inputs for better tailoring vaccination campaigns.

## 1. Introduction

Health inequalities are the unjust and avoidable differences in people’s health across the population and among specific population groups. The existence of health inequalities means that the right of everyone to the highest attainable standard of health is not being enjoyed equally across the population [1].

COVID-19 seems to have amplified health inequalities. The UK Office for National Statistics published a report about COVID-19 mortality rates according to level of deprivation, which shows a clear social gradient with the more deprived areas having higher mortality [2]. Also, the risk of infection is up to three times greater in very high-deprivation areas compared to very low-deprivation areas. Risks of hospitalization and testing also increase with deprivation, but to a lesser extent [2,3].

Social determinants of health, namely, “the conditions in which people are born, grow, live, work and age”, are nonmedical factors that commonly lead to inequalities in health status and in the use of health services and access to affordable health services [4,5].

In the Umbria Region, as well as in the rest of Italy, despite a universal Regional Health Service that aims to provide all with the same health services, socio-economic inequalities in access to health care and services have been reported [6,7].

Among health services, also vaccination might be impacted by social determinants and show access inequalities. The literature reported that factors such as geographic ones (geographic area and following residence and area-level deprivation) and socio-demographic ones (income, social class, marital status, education, foreign citizenship, and sex) can affect vaccination access and uptake [8,9,10,11,12,13].

In Italy, COVID-19 vaccines were offered to healthcare workers first and to the general population after, following priority criteria based on individual vulnerability. Eventually, as of December 2021, all the adult population who wanted to vaccinate against COVID-19 had the possibility of being fully vaccinated.

A comprehensive COVID-19 vaccination campaign seemed able to reduce socio-economic inequalities; however, several studies investigated the characteristics associated with the willingness to receive the vaccination against COVID-19 showing greater hesitancy in the disadvantaged groups of the population [14,15,16,17].

A recent review demonstrated that, in Italy, hesitancy towards COVID-19 vaccination was influenced by various factors, particularly related to misinformation, perceived efficacy, and safety of vaccines, but also social determinants, such as age, citizenship, residence, educational level, occupation and sex [18]. Hesitancy towards COVID-19 vaccination has been mostly evaluated through the measurement of intention/willingness to vaccinate in population samples.

On the contrary, few studies have relied on the measurement of the real uptake of vaccination in whole populations, and only part of them considered the overall population [19,20,21,22,23].

The present study aimed to analyze COVID-19 vaccination uptake in the overall population of the Umbria Region and to identify potential inequalities associated with socio-demographic factors through a population-based study.

## 2. Materials and Methods

### 2.1. Study Design and Setting

We conducted a population-based study to analyze the socio-demographic factors associated with nonvaccination against COVID-19 as of 28 February 2022.

The study was conducted in the Umbria Region of central Italy, which had a population of approximately 859,000 residents in January 2022 [24]. The Regional Health Service provides comprehensive health care to the resident population. The COVID-19 vaccination campaign in the Umbria Region started on 27 December 2020, with approximately 3.3% of the resident population having received a diagnosis of SARS-CoV-2 infection at that date. Healthcare workers and nursing home residents were the first to get vaccinated, through dedicated vaccination points. Afterward, around mid-February 2021, territorial vaccination points were opened and the vaccination started to be offered to the entire population, according to national priority groups indications [25]. Finally, within one year from the opening of territorial vaccination points, as of 28 February 2022, the entire vaccinable population could have received at least one dose of a COVID-19 vaccine, and adults could have completed the initial vaccination schedule and obtained a booster dose as well.

### 2.2. Study Population

The population included in the study was represented by the resident population registered in the Regional Health Service as of 28 February 2021 (N = 866,678). From this population, eligible subjects for vaccination were selected (N = 838,043), excluding children under 5 years of age and those who had obtained a certificate of COVID-19 vaccination exemption up to 28 February 2022.

**Figure 1:**
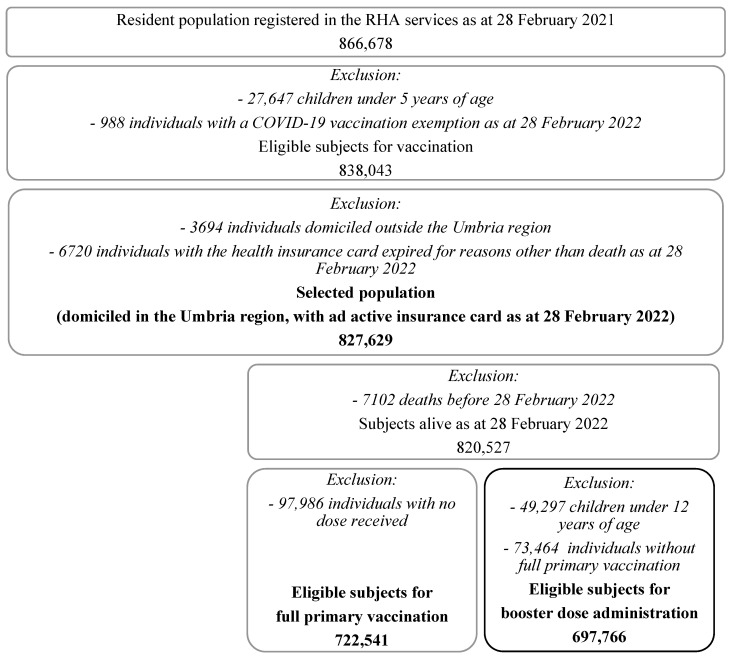
Study population selection for primary and secondary endpoints.

In order to avoid a potential information bias due to vaccinations administered outside the region—with these data possibly not being recorded in the regional registers—we also excluded individuals domiciled outside the region and those whose health insurance card had expired for reasons other than death as of 28 February 2022, for a final number of 827,629 selected individuals with accessible, complete and reliable data on vaccination status (Figure 1).

For the assessment of vaccination status in respect to the doses following the first one, in order to correctly evaluate data about the completion of the full primary vaccination cycle, we first selected 820,527 individuals alive as of 28 February 2022; then, subjects eligible for completing full primary vaccination were selected excluding individuals with no dose received. In respect to the booster dose, all individuals under 12 years of age, for whom the booster dose was not planned as of 28 February 2022, were excluded as well as individuals who did not complete the full primary vaccination cycle (Figure 1).

### 2.3. Data Source

We linked the Regional Health Information System with the DBCOVID Umbria regional database, using citizens’ regional unique identification codes. The Regional Health Information System contains all regional health administrative databases, with anagraphic data of population assisted by the Health Service and information on General Practitioners (GPs) or Family Pediatricians (FPs) and on exemptions. The DBCOVID Umbria regional database is a longitudinal database that has collected individual data of the regional SARS-CoV-2 Integrated Surveillance System since February 2020; it contains data about SARS-CoV-2 testing results and immunization. The data management and analysis were carried out at the Epidemiology Service of the Prevention Department of the Local Health Unit 1 of Umbria Region, which guaranteed its processing in compliance with privacy regulations. The study was conducted in accordance with the Declaration of Helsinki, and approved by the Regional Ethics Committee of Umbria, Italy “CER Umbria” (CER N 4183/19, protocol code 23155/21/ON; date of approval: 27/10/2021).

### 2.4. Study Endpoints and Variables 

Nonadherence to COVID-19 vaccination as of 28 February 2022 was considered as the primary endpoint, with adherence being defined as the administration of at least one dose of any COVID-19 vaccine.

The failure to complete the full primary vaccination, namely, the uptake in various possible combinations of two doses of Pfizer-BioNTech, Moderna or Vaxzevria vaccines, or of a single dose of Johnson & Johnson, or of a single dose of any vaccine within one year of the SARS-CoV-2 infection (previous or subsequent), was considered as the secondary endpoint. Failure to get the booster dose in the eligible population was also considered as a secondary endpoint. Possible delays in uptake due to SARS-CoV-2 infections were not considered when assessing the outcomes.

As potential factors associated with the primary and secondary endpoints, the following individual-level variables were considered based on their availability, completeness, reliability and potential usefulness for informing future vaccination campaigns:

- sex (male or female);
- age (5–11, 12–19, 20–29, 30–39, 40–49, 50–59, 60–69, 70–79, 80–89, 90+);
- citizenship (Italian or non-Italian);
- holding of an officially recognized exemption—that allows citizens to obtain total or partial exemption from paying the health ticket—due to specific health conditions, i.e., chronic/rare pathologies or disabilities, used as a proxy for frailty (present or absent) [26,27];
- holding of a General Practitioner (GP) or a Family Pediatrician (FP) who is commonly chosen by each resident to obtain most of the primary care (present or absent).

We also extracted data about municipality of residence (92 municipalities) that were then used to associate citizens to the deprivation index measured at the municipalities level, according to the Italian National Deprivation Index [28]. The latter was a composite indicator based on 5 variables (low level of education, unemployment, non-home ownership, one-parent family and overcrowding) derived from the 2011 Italian census data and categorized in quintiles—from most (group 5) to least (group 1) deprived—weighted by the regional population.

### 2.5. Statistical Analyses

The study population was first described overall and according to primary and secondary endpoints, using frequencies and percentages for categorical variables and mean ± standard deviation (SD) for quantitative variables. Logistic regression models were used to study factors associated with nonadherence to vaccination, failure to complete the full primary vaccination and get the booster dose. Univariable logistic models were first performed (Supplementary Materials—Table S1), and all variables were shown to be significantly related to all the endpoints; hence, all variables were included in the final fully adjusted model. Odds ratios (ORs) and associated 95% confidence intervals (95% CIs) were considered to evaluate the strength of the associations. We accounted for the clustering of individuals within municipalities obtaining a robust variance estimate that adjusts for within-cluster correlation. A variance inflation factor (VIF) was calculated for each covariate included in the models, detecting low evidence of collinearity (VIF < 4 for all covariates). Analyses were also stratified by sex. Statistical significance was set at *p* < 0.05. All analyses were performed with the statistical software Stata 14.0 (StataCorp LLC, College Station, TX, 77845, USA).

## 3. Results

As of 28 February 2022, out of 827,629 vaccinable individuals (48.1% males), 11.8% did not uptake COVID-19 vaccination (Table 1). Among those who initially took part in the vaccination campaign (at least one dose of vaccine administered, N = 729,630) and did not die within a year (N = 722,541), 1.2% (N = 8651) had failed to complete the full primary vaccination cycle, and 21.5% (N = 150,033) of those eligible for booster dose administration (N = 697,766) did not receive it (Table 1).

The percentage of nonadherent subjects was similar in men and women (11.7% vs. 12.0%), higher in non-Italians than in Italians (26.1% vs. 10.3%) and in those who had not been exempted compared to those who had an exemption for a chronic or rare pathology or for invalidity due to medical causes (14.4% vs. 5.7%); moreover, the percentage of nonadherent subjects decreased with increasing age, with the lowest values among the age group 80–89 (4.2%), even though the percentage of nonadherent people slightly increased among the 90+ age group (6.7%). Among those who had not had a GP or FP in the last two years, the percentage of nonadherent people reached 47.0%. The percentage of nonadherent people was slightly similar across quintiles of the deprivation index.

With regard to the failure to complete the full primary vaccination and get the booster dose, the findings were almost similar with a decline of the gap between subjects with and without a GP/FP for the failure to uptake the booster dose.

The final fully adjusted logistic model on the nonadherence to vaccination (Table 2) showed that women had a significant 4.2% higher probability to not vaccinate themselves than men. Nonadherence to vaccination was inversely associated with increasing age, even though, taking the 80–89 age group as a reference, a trend inversion was observed in the 90+ age group. Non-Italian citizens, as compared to Italians, showed a significant 188% increased likelihood to not uptake vaccination. Subjects with better health conditions had a significant 43.2% higher probability to not vaccinate themselves than subjects with exemptions for pathologies or disability. The factor more strongly associated with nonadherence appeared to be the lack of a GP/FP (OR: 8.91). Sex-disaggregated analysis showed that the association with age was more pronounced among males than among females, whereas the one with citizenship was more pronounced in females than in males.

The analysis of the failure to complete the full primary vaccination cycle showed similar results even though associations were less strong as compared to the results on the primary endpoint and disappeared relative to sex. The same can be concluded for the analysis on the failure to get the booster dose except for sex, which did not show any association with the completion of full primary vaccination cycle and issued a protective role of female sex regarding the failure of getting the booster dose. Of note, the role of the presence/absence of a GP/FP was significantly scaled down in the analysis of secondary endpoints, i.e., the continuation of the vaccination course (Table 3). Since there was no evidence of an effect modification by sex, we did not report sex-disaggregated results for secondary endpoints.

## 4. Discussion

This paper provided an overview of COVID-19 vaccination initiation and completion and associated socio-demographic factors on the whole population of the Umbria Region.

Overall, the uptake of the first dose was around 90% and primary vaccination cycle completion was very high, as only 1.2% of people who initiated the vaccination did not complete it. On the contrary, the uptake of the booster dose was slightly below 80%. These data are anyway aligned with nationwide statistics on COVID-19 vaccination coverage in Italy [29], but it is necessary to point out that they can slightly deviate from those publicly available on the Umbrian dashboard because of the selection criteria used to identify the study population and the different denominators used to calculate coverages.

The analysis of the association between socio-demographic characteristics and the nonadherence to vaccination revealed that females were less likely to initiate vaccination but less likely to fail to get the booster dose. Younger people, non-Italian citizens, those without exemptions for disability/chronic diseases and without a GP/FP were more likely to neither initiate nor complete the vaccination cycle. On the contrary, results about the role of deprivation index were not conclusive, even though a reduction in nonadherence to vaccination as well as in the failure to get the full cycle and the booster dose was observed with the increase in deprivation. These results are overall aligned with those coming from a similar population-based study performed in the Lazio Region that also analyzed the education level, showing an association between low educational level and the chance of not getting the vaccination [21]. The same study also found a relationship between deprivation index and vaccination uptake with an increasing likelihood of being unvaccinated passing from less deprived to most deprived areas. Moreover, the study performed in the Metropolitan Area of Milan [22] issued the same results compared to ours in respect to the role of gender, citizenship and chronic conditions. It also showed that people living in more deprived areas were less likely to be adherent and that younger people were more adherent. Nevertheless, these two studies, which were conducted in a similar way, did not make a separate analysis on the uptake of the first dose, the completion of the full vaccination cycle and the uptake of the booster dose. This is one of the interesting and novel aspects addressed by our work alongside the holding of a GP/FP that was not addressed in previous works.

The relationship between sex, age and uptake of vaccination can be discussed in the light of current knowledge on the factors influencing vaccine hesitancy. In fact, two systematic reviews on COVID-19 vaccination hesitancy in the Italian population reported a higher hesitancy in females and younger people [18,30]. These results were also confirmed by studies performed outside Italy and their systematic reviews [31,32]. A systematic review of reviews confirmed these associations [33] and also reported a relationship between hesitancy and social determinants, such as non-white ethnicity, lower education, income level and living conditions. This evidence is important to elaborate on the results on citizenship and deprivation that were issued by our study. In respect to citizenship, the higher probability of being nonadherent that was observed in non-Italian citizens could be due to the presence, among them, of Black, Asian and minority ethnic groups that have been already documented to be more hesitant than white people [33]. Nevertheless, the result can be also justified by other aspects linked to the ease of access to health services. Of note, our study also highlighted that not holding a GP/FP increases the risk of neither initiating nor completing the vaccination. Non-Italian people in Italy can be registered with the National Health Service and receive assistance under the same conditions as Italian citizens, including holding a GP/FP. The small share of people included in our study who did not hold a GP/FP and showed the highest probability to not uptake the vaccination could be represented by a subgroup of the population with specific characteristics that need to be better investigated in future research. Our results on holding a GP/FP and its relationship with vaccination uptake might be reasoned by the role of a GP/FP as patients’ first contact to the healthcare system and in guiding patients’ choices. As a matter of fact, GP/FP are reported as the most common source that people use to obtain information on vaccines/vaccination [34]; therefore, his/her absence could be responsible for a lack of knowledge that could have impacted the final choice to get vaccinated.

In respect to deprivation, the Italian index used in this study considered factors that have been already shown to be associated with COVID-19 vaccine hesitancy [33], namely, low level of education, unemployment, non-home ownership, one-parent family and overcrowding [28]. The role of deprivation has already been investigated in the field of vaccination, and the evidence showed that most deprived areas as well as lower socio-economic groups are generally at higher risk of incomplete/delayed vaccination and generally show lower vaccination coverage [35,36]. Our study missed to show an association between vaccination initiation/completion and deprivation status, but other population-based studies that addressed community characteristics, including deprivation, showed that most deprived areas and most ethnically diverse areas were at higher risk of lower COVID-19 vaccine uptake [21,22,37,38]. The different results issued by our and similar studies conducted in Italy [21,22] could be attributed to the fact that we did not obtain the information on the deprivation index according to the census tract but to the municipality, thus lacking a more granular insight. This could have led to less variability and, therefore, to a loss of power in detecting differences. Because of these reasons, the findings on the deprivation index should be considered with caution. Finally, in respect to underlying chronic conditions/disability, the results of our study were expected in respect to the available literature that already showed the association between the presence of comorbidities and the vaccine uptake [39], even though the presence of chronic conditions could have been underestimated in our study because of the choice of relying on administrative exception. This finding is also consistent with how the vaccination campaign has been structured, with prioritization and particular attention to frail people.

## 5. Conclusions

Based on our results, it can be concluded that socio-demographic characteristics are associated with vaccine initiation and completion and may contribute to inequalities in vaccine uptake. In particular, age, sex, citizenship, and holding an exemption for disability/chronic diseases and a GP/FP were all associated with vaccine uptake. This conclusion could be helpful to better and further tailoring current and future vaccination campaigns, as socio-demographic data are easily accessible and analyzable on the contrary of other factors that are associated with vaccine hesitancy but are not promptly available, such as individual and group influences and vaccine and vaccination specific influences. The latter, as well as other aspects that might influence the access to vaccination services and determine potential inequalities, such as organizational ones, were not investigated in our study, and this represents one of the main limitations. Another limitation is linked to the fact that we could not address delay in vaccination completion and receipt of the booster dose due to any reason (adverse events, type of vaccines received, data flow among different informatics systems). This was not possible because the study relied on available region-wide data flows that catch only a part of information. Furthermore, the types of vaccines available in different times and territories or provided to different categories of people could have affected the vaccination completion, due to unequal adverse events and their media resonance. A further limitation is represented by the use of citizenship as an alternative to birthplace that is often used in the literature to define the migrant status. Nevertheless, this more restrictive criterion that we used could catch immigrants who are unlikely to have the same legal rights as citizens in the host country and more likely to face economic and social difficulties. Furthermore, not obtaining the information on the deprivation index to the census tract could have led to a loss of power in detecting differences. Eventually, the use of administrative data sources might have also brought to the underestimation of people having chronic conditions. Nevertheless, to the best of our knowledge, this work is one of the few population-based studies, not based on survey data, that has been performed in Italy to investigate the role of socio-demographic factors in COVID-19 vaccination uptake and the first to disentangle this issue in respect to both the initiation and completion of COVID-19 vaccination. The reliability of data source, the carefully exclusion of people with potential incomplete or nonsense data (i.e., those domiciled outside the Umbria Region, not registered with the Regional Health Service, and those who died after receiving the first dose) and the choice of a robust outcome, namely, the actual uptake of vaccination, made it possible to draw solid conclusions and reinforce and advance the knowledge on this topic in the light of informing future vaccination campaigns.

## Figures and Tables

**Table 1:** Characteristics of the Umbrian population eligible for vaccination and percentages of primary and secondary endpoints as of 28 February 2022.

Characteristics	Vaccinable		Not Adherent to Vaccination Campaign (Any Dose) (N = 827,629)	Failed to Complete the Full Primary Vaccination (N = 722,541)	Failed to Get the Booster Dose (N = 697,766)
	N	%	%	%	%
Total	827,629	100.0	11.8	1.2	21.5
Sex					
Males	397,820	48.1	11.7	1.21	22.3
Females	429,809	51.9	12.0	1.18	20.8
Age					
Average (SD)	49.3 (22.9)		33.7 (24.2)	32.7 (24.2)	40.0 (19.4)
5–11	49,297	6.0	62.3	13.4	-
12–19	62,983	7.6	14.0	2.3	51.8
20–29	77,340	9.3	9.6	1.3	37.1
30–39	89,244	10.8	11.9	1.0	31.3
40–49	119,335	14.4	10.6	0.7	24.6
50–59	135,107	16.3	8.4	0.9	18.2
60–69	113,374	13.7	7.0	0.6	11.0
70–79	96,886	11.7	4.7	0.4	8.3
80–89	65,991	8.0	4.2	0.5	6.6
90+	18,072	2.2	6.7	0.8	9.2
Citizenship					
Italian	747,528	90.3	10.3	1.1	19.8
Non-Italian	80,101	9.7	26.1	2.8	41.4
Comorbidity/Disability *					
Yes	242,108	29.3	5.7	0.6	12.5
No	585,521	70.7	14.4	1.5	25.8
GP/FP					
Yes	818,101	98.8	11.4	1.2	21.4
No	9528	1.2	47.0	4.3	30.6
Deprivation					
1 quintile	167,221	20.2	12.1	1.3	21.2
2 quintile	160,686	19.4	11.4	1.1	20.5
3 quintile	185,307	22.4	12.1	1.2	22.0
4 quintile	155,285	18.8	10.9	1.1	21.2
5 quintile	159,130	19.2	12.7	1.2	22.6

* based on officially recognized health ticket exemptions due to a chronic/rare pathology or a disability.

**Table 2:** Characteristics associated with nonadherence to vaccination campaign (primary endpoint); Umbria Region as of 28 February 2022.

	Not Adherent to the Vaccine Campaign (Any Dose)					
	All (N = 827,629)		Males (N = 397,820)		Females (N = 429,809)	
Characteristics	Fully Adj. OR	95% CI	Fully Adj. OR	95% CI	Fully Adj. OR	95% CI
Sex						
Males	(Reference)					
Females	**1.042**	1.030–1.054				
Age						
5–11	**29.571**	**27.015–32.369**	**39.681**	**35.990–43.752**	**24.910**	**22.542–27.527**
12–19	**2.740**	**2.534–2.962**	**3.757**	**3.420–4.127**	**2.258**	**2.079–2.453**
20–29	**1.689**	**1.573–1.814**	**2.279**	**2.109–2.463**	**1.416**	**1.294–1.549**
30–39	**2.034**	**1.926–2.148**	**2.794**	**2.593–3.010**	**1.670**	**1.575–1.771**
40–49	**1.916**	**1.801–2.038**	**2.736**	**2.527–2.963**	**1.513**	**1.407–1.626**
50–59	**1.655**	**1.569–1.745**	**2.216**	**2.057–2.388**	**1.390**	**1.310–1.475**
60–69	**1.510**	**1.436–1.589**	**1.872**	**1.749–2.002**	**1.353**	**1.274–1.437**
70–79	**1.096**	**1.038–1.158**	**1.247**	**1.156–1.345**	1.045	0.977–1.118
80–89	(Reference)		(Reference)		(Reference)	
90+	**1.662**	**1.543–1.790**	**1.588**	**1.375–1.834**	**1.585**	**1.465–1.714**
Citizenship						
Italian	(Reference)		(Reference)		(Reference)	
Non-Italian	**2.878**	**2.678–3.093**	**2.642**	**2.478–2.816**	**3.092**	**2.839–3.368**
Comorbidity/Disability *						
Yes	(Reference)				(Reference)	
No	**1.432**	**1.378–1.490**	**1.457**	**1.396–1.520**	**1.406**	**1.349–1.465**
GP/FP						
Yes	(Reference)				(Reference)	
No	**8.919**	**7.731–10.289**	**8.565**	**7.324–10.017**	**9.391**	**8.036–10.975**
Deprivation						
1 quintile	(Reference)		(Reference)		(Reference)	
2 quintile	0.902	0.773–1.053	0.913	0.780–1.067	0.893	0.764–1.043
3 quintile	0.938	0.816–1.079	0.941	0.819–1081	0.935	0.810–1.079
4 quintile	**0.791**	**0.719–0.870**	**0.799**	**0.732–0.873**	**0.781**	**0.704–0.867**
5 quintile	1.021	0.873–1.194	1.034	0.880–2.215	1.009	0.865–1.177

* based on officially recognized health ticket exemptions due to a chronic/rare pathology or a disability; significant results are reported in bold.

**Table 3:** Characteristics associated with failure to complete the full primary vaccination and failure to get the booster dose (secondary endpoints); Umbria Region as of 28 February 2022.

	Failure to Complete the Primary Vaccination Cycle (N = 722,541)		Failure to Get the Booster Dose (N = 697,766)	
Characteristics	Fully Adj. OR	95% CI	Fully Adj. OR	95% CI
Sex				
Males	(Reference)		(Reference)	
Females	1.030	0.987–1.075	**0.968**	**0.957–0.980**
Age				
5–11	**29.414**	**25.239–34.279**	-	-
12–19	**4.123**	**3.563–4.772**	**12.769**	**12.175–13.392**
20–29	**2.238**	**1.941–2.581**	**6.883**	**6.367–7.441**
30–39	**1.597**	**1.334–1.912**	**5.160**	**4.859–5.479**
40–49	**1.225**	**1.028–1.460**	**3.805**	**3.621–3.998**
50–59	**1.595**	**1.351–1.882**	**2.733**	**2.600–2.873**
60–69	1.145	0.964–1.360	**1.618**	**1.547–1.694**
70–79	0.931	0.788–1.101	**1.248**	**1.196–1.302**
80–89	(Reference)		(Reference)	
90+	**1.834**	**1.469–2.290**	**1.474**	**1.384–1.569**
Citizenship				
Italian	(Reference)		(Reference)	
Non-Italian	**2.732**	**2.588–2.884**	**2.201**	**2.039–2.375**
Comorbidity/Disability *				
Yes	(Reference)		(Reference)	
No	**1.139**	**1.071–1.211**	**1.227**	**1.208–1.246**
GP/FP				
Yes	(Reference)		(Reference)	
No	**4.771**	**3.968–5.738**	**1.487**	**1.352–1.635**
Deprivation				
1 quintile	(Reference)		(Reference)	
2 quintile	**0.840**	**0.747–0.943**	0.943	0.873–1.018
3 quintile	0.933	0.841–1.035	1.016	0.940–1.098
4 quintile	**0.758**	**0.696–0.825**	**0.916**	**0.873–0.961**
5 quintile	0.912	0.825–1.007	1.052	0.994–1.114

* based on officially recognized health ticket exemptions due to a chronic/rare pathology or a disability; significant results are reported in bold.

## Data Availability

The data presented in this study are available on request from the corresponding author. The data are not publicly available due to privacy issues.

## Supplementary Materials

The following supporting information can be downloaded at: https://www.mdpi.com/article/10.3390/vaccines11081351/s1, Table S1: Characteristics associated with non-adherence to vaccination campaign (primary endpoint) and with failure to complete the full primary vaccination and failure to get the booster dose (secondary endpoints); univariate models; Umbria Region as of 28 February 2022.
