# Supplementary material for: COVID-19 Vaccination Actual Uptake and Potential Inequalities Due to Socio-Demographic Characteristics: A Population-Based Study in the Umbria Region, Italy
Source: Vaccines (Basel). 2023 Aug 9;11(8):1351. doi: 10.3390/vaccines11081351 (PMC10458483; doi:10.3390/vaccines11081351)
Supplement: Supplementary file 1 [file vaccines-11-01351-s001.zip › vaccines-2507965-supplementary.pdf]

Table S1. Characteristics associated with non-adherence to vaccination campaign (primary endpoint) and with failure to complete the full primary vaccination and failure to get the booster dose (secondary endpoints); univariate models; Umbria Region as of February 28th, 2022.

	Not adherent to the vaccine campaign (any dose)		Failure to complete the primary vaccination cycle (N=722,541)		Failure to get the booster dose (N=697,766)	
**Characteristics**	**OR**	**95% C.I.**	**OR**	**95% C.I.**	**OR**	**95% C.I.**
**Sex**						
Males	(Reference)		(Reference)		(Reference)	
Females	**0.979**	**0.966-0.992**	**0.976**	**0.935-1.019**	**0.917**	**0.904-0.930**
**Age**						
5-11	**37.924**	**34.680-41.471**	**33.428**	**28.555-39.131**	**-**	**-**
12-19	**3.741**	**3.460-4.045**	**5.069**	**4.377-5.871**	**15.163**	**14.439-15.924**
20-29	**2.444**	**2.220-2.692**	**2.864**	**2.466-3.326**	**8.346**	**7.641-9.115**
30-39	**3.091**	**2.878-3.321**	**2.150**	**1.785-2.591**	**6.441**	**6.012-6.900**
40-49	**2.711**	**2.521-2.915**	**1.573**	**1.305-1.896**	**4.618**	**4.375-4.875**
50-59	**2.119**	**1.997-2.249**	**1.899**	**1.606-2.246**	**3.143**	**2.981-3.313**
60-69	**1.737**	**1.637-1.843**	**1.261**	**1.058-1.504**	**1.746**	**1.665-1.830**
70-79	**1.141**	**1.078-1.207**	**0.958**	0.811-1.132	**1.277**	**1.224-1.332**
80-89	(Reference)		(Reference)		(Reference)	
90+	**1.662**	**1.542-1.791**	**1.817**	**1.457-1.132**	**1.439**	**1.352-1.531**
**Citizenship**						
Italian	(Reference)		(Reference)		(Reference)	
Non Italian	**3.065**	**2.881-3.260**	**2.654**	**2.523-2.792**	**2.870**	**2.693-3.059**
**Comorbidity/Disability***						
Yes	(Reference)		(Reference)		(Reference)	
No	**2.798**	**2.681-2.919**	**2.330**	**2.151-2.524**	**2.434**	**2.342-2.530**
**GP/FP**						
Yes	(Reference)		(Reference)		(Reference)	
No	**6.876**	**5.995-7.887**	**3.757**	**3.205-4.405**	**1.616**	**1.415-1.847**
**Deprivation**						
1 quintile	(Reference)		(Reference)		(Reference)	
2 quintile	**0.93**	**0.914-0.954**	**0.869**	**0.812-0.929**	**0.959**	**0.942-0.977**
3 quintile	1.000	0.980-1.021	0.952	0.894-1.015	**1.045**	**1.028-1.064**
4 quintile	**0.892**	**0.873-0.911**	**0.855**	**0.799-0.915**	0.996	0.978-1.015
5 quintile	**1.053**	**1.032-1.076**	**0.924**	**0.865-0.988**	**1.081**	**1.061-1.100**

* based on officially recognized exemptions due to a chronic/rare pathology or a disability; significant results are reported in bold.
